# Potential and Challenges in Collecting Social and Behavioral Data on Adolescent Alcohol Norms: Comparing Respondent-Driven Sampling and Web-Based Respondent-Driven Sampling

**DOI:** 10.2196/jmir.4762

**Published:** 2015-12-24

**Authors:** Janina Hildebrand, Sharyn Burns, Yun Zhao, Roanna Lobo, Peter Howat, Steve Allsop, Bruce Maycock

**Affiliations:** ^1^ Collaboration for Evidence, Research and Impact in Public Health School of Public Health Curtin University Bentley Australia; ^2^ School of Public Health Curtin University Bentley Australia; ^3^ National Drug Research Institute Curtin University Perth Australia

**Keywords:** Internet, respondent-driven sampling (RDS), WebRDS, adolescent, alcohol, social media, participant recruitment

## Abstract

**Background:**

Respondent-driven sampling (RDS) is a method successfully used to research hard-to-access populations. Few studies have explored the use of the Internet and social media with RDS, known as Web-based RDS (WebRDS). This study explored the use of combining both “traditional” RDS and WebRDS to examine the influences on adolescent alcohol use.

**Objective:**

This paper reports on the recruitment processes and the challenges and enablers of both RDS and WebRDS. It details comparative recruitment data and provides a summary of the utility of both methods for recruiting adolescents to participate in an online survey investigating youth alcohol norms.

**Methods:**

Process evaluation data collected from research staff throughout the study were used to assess the challenges and solutions of RDS and WebRDS. Pearson chi-square test (Fisher’s exact test if applicable) was used to compare the differences in sociodemographics and drinking behavior between data collected by RDS and WebRDS.

**Results:**

Of the total sample (N=1012), 232 adolescents were recruited by RDS and 780 by WebRDS. A significantly larger proportion of Aboriginal or Torres Strait Islander (*P*<.001) participants who spoke English as their main language at home (*P*=.03), and of middle and lower socioeconomic status (*P*<.001) was found in the RDS sample. The RDS sample was also found to have a higher occurrence of past 7-day drinking (*P*<.001) and past 7-day risky drinking (*P*=.004). No significant differences in gender, age, past month alcohol use, and lifetime alcohol use were observed between the RDS and WebRDS samples. This study revealed RDS and WebRDS used similar lengths of chains for recruiting participants; however, WebRDS conducted a faster rate of recruitment at a lower average cost per participant compared to RDS.

**Conclusions:**

Using WebRDS resulted in significant improvements in the recruitment rate and was a more effective and efficient use of resources than the traditional RDS method. However, WebRDS resulted in partially different sample characteristics to traditional RDS. This potential effect should be considered when selecting the most appropriate data collection method.

## Introduction

Research has shown that respondent-driven sampling (RDS) is a viable method to recruit individuals from hard-to-access populations (eg, drug users and sex workers) for which no sampling frame exists [1-8]. It has also been used successfully to examine young people’s risk-taking behavior [9-12] in several countries [9,10,13,14] and settings [11,12,14]. RDS is a probability sampling method, which is a modified form of chain referral sampling [15]. RDS uses chain referrals with structured incentives as a recruitment strategy, whereby social network parameters of participants are used to weigh the data to statistically adjust for potential chain referral bias [16]. Samples generated by RDS are generally more heterogeneous than those recruited via other sampling means, thus are potentially more generalizable to the population of interest [8,17,18]. In addition, RDS allows validity and reliability of results and randomization of the sample [13]. RDS is not constrained by certain biases associated with other snowball-type recruitment methods, such as time-space for which the sample is restricted to individuals present in public venues [15,19]. Nonprobability snowball sampling is a nonrandom convenience method from which biased estimation is likely to occur [20]. In contrast, the aforementioned characteristics of RDS enable better representation of social networks and consequently more valid calculation of population estimates [16]. Limitations of RDS include the potentially high cost of data collection, requiring substantial resources in terms of personnel and cost [21]. Zablotska et al [22], in their comparison of data collection methods, concluded that their RDS sample was the most consistent to population estimates, but that it was complex and logistically demanding compared to time-location and online recruitment, which were more cost-effective and easier to implement. RDS estimates have also shown a larger variance compared to simple random sampling [23]. For these reasons, Goel and Salganik [23] suggested RDS may not be an optimal strategy for public health surveillance.

Recently, RDS has adopted Web-based recruitment methods (WebRDS) [21,24]. Two studies reported that WebRDS may be a feasible alternative to traditional RDS in the recruitment of young adults [21,24]. Bauermeister et al [21] used WebRDS to administer an online survey designed to assess alcohol and other drug use among those aged 18 to 24 years in the United States. Study primary participants (seeds) were recruited online through a targeted Facebook advertisement. Compared with traditional RDS or face-to-face recruitment, WebRDS demonstrated an ability to overcome temporal and physical barriers to recruitment by allowing young adults to refer a peer using features on social networking sites, such as a wall post, status update, or personal message. Offering multiple approaches to peer referral was found to be most effective in maximizing rate of data collection and length of referral chains, the total number of waves recruited by a seed. Limitations to achieving a representative sample in this study included racial/ethnic and socioeconomic disparities in computer access and frequency of use. Particularly, low education levels and self-identified nonwhite individuals were underrepresented in the sample [21].

Wejnert and Heckathorn [24] also explored the use of WebRDS to obtain a sample of 159 American undergraduate students, initiated from 9 seeds recruited via the Internet. Their findings support the potential for WebRDS to generate electronic network chains with minimal resources and at a significantly faster rate than traditional RDS methodology. Their study also recommended using small incentives due to the reduced respondent burden associated with WebRDS. Although preliminary results indicate WebRDS may be a more efficient recruitment method compared with traditional RDS and standard sampling strategies [25], there is a dearth of studies using this method in an adolescent population.

This paper describes the adaptive and iterative methods from a study using RDS and WebRDS to recruit a sample of youth aged 14 to 17 years exploring alcohol-related norms and behaviors using an online survey. This paper details the recruitment processes and the challenges and enablers of both RDS and WebRDS and compares recruitment data and the utility of both methods for recruiting and collecting data from adolescents.

## Methods

Over a 21-week period, a combination of RDS and WebRDS was used to recruit adolescents aged 14 to 17 years living in Perth, Western Australia, to complete an online survey investigating their alcohol-related social norms and behaviors [26]. The comprehensive survey instrument was substantially longer than those of other WebRDS studies [21,24] and, therefore, had higher potential risk of participant attrition. Challenges and solutions of each recruitment method are discussed subsequently.

Initial study participants using an RDS approach (seeds) were purposively selected from the community, youth programs, and sports clubs. Study eligibility was confirmed face-to-face by a trained staff member through a series of screening questions to determine age (14-17 years), location (Perth metropolitan area), and previous study involvement. The use of WebRDS evolved as a response to the barriers experienced during the traditional RDS recruitment.

### Procedure

RDS was conducted face-to-face with participants meeting with a staff member to complete the survey on a tablet device (iPad) or a paper version. The seeds, and their subsequent referrals, were given referral coupons and asked to recruit up to 3 of their peers into the study within 2 weeks of their survey completion. Participants received an AU $15 gift card to an electronics store for completing the survey. For each subsequent successful referral, they received an additional AU $10 gift card for a possible total incentive of AU $45. To meet ethics requirements, each participant completed a mature minor assessment and provided verbal and written informed consent to participate [27]. Participants could cease involvement at any point. See Hildebrand et al [26,28] for full descriptions of RDS methodology and this study’s survey procedure.

The mature minor assessment was undertaken by providing potential participants with a verbal and written explanation of the study, explaining the purpose of the study, who was conducting it and the anticipated outcomes, and the requirements of participation. Participant information sheets were pilot-tested with members of the target group and assessed for readability to ensure a reading age of 11 years (grade 6). It was explicitly stated that the decision to participate in the research was that of the young person’s alone and that they could withdraw at any time without prejudice. Adolescents were given the opportunity to ask questions along with sufficient time to make a decision. A protocol checklist to establish each adolescent’s ability to understand the requirements of the study and their mature minor status before deciding to participate was developed. Using the protocol checklist, cognitive interviewing was adapted and applied to evaluate each participant’s comprehension of the study information and consent materials. Potential participants were asked a set of 5 questions pertaining to the study procedures, which they had to answer verbally. Participants who provided verbal response to the questions that demonstrated their understanding of the ethical considerations and participant rights, were assigned mature minor status and were considered able to provide consent and continue participation. Data collection officers completed training, which included explanation of the process, the importance of consistency, opportunity to practice the procedure, and a manual outlining the steps and a set of required responses. The project manager met with the data collection officers on a weekly basis to monitor and collect feedback on the progress [27].

### Challenges and Solutions of Respondent-Driven Sampling

Throughout the data collection, we faced a number of challenges due to the age of the target population and implications of adhering to the specific RDS methodology and ethical considerations [27]. The greatest challenge encountered was lack of use of the referral coupons. Only 2 participants initiated contact with the study staff from these coupons. Forty-three participants who had conducted the survey face-to-face were contacted. Feedback on attempts to distribute referral coupons indicated that many participants had not attempted to recruit peers. Some reasons cited for not distributing coupons were forgetting, loss of the coupon, going on holiday, all friends being involved in the initial recruitment, being too busy to pass on, friends being too busy to be involved, and thinking friends would not be interested. Overall, reliance on paper coupons to facilitate referrals did not result in an adequate referral rate or chain length.

Additional resources were allocated to enhance recruitment, including presentations conducted at the beginning of sports training or youth program sessions and increased focus on locations to administer surveys “on the spot” where groups of youth congregated (shopping centers, movie theaters, and the central business district). Referrals were sought and immediately recruited if they were part of the existing group by asking participants if they would invite any of their peers who were present as their referrals. The best opportunities to approach and recruit participants were found to be when youth were spending time with friends, such as after finishing lunch in a food court or while waiting outside a theater for a movie to start, and not engaged in other activities.

Participants recruited from youth programs posed a particular challenge for data collection. Feedback from youth program staff and data collection officers suggest issues such as low literacy levels, participants skipping questions, or not having access to Internet or a personal email account to enable follow-up made data collection difficult. Although these challenges were encountered generally among youth, they were particularly common among at-risk youth. Even when a mobile phone number was provided, there were issues associated with participants running out of phone credit. Participants from youth group programs also commonly claimed to have no relevant peers to refer.

### Web-Based Respondent-Driven Sampling

During recruitment, a large proportion of participants indicated that their preferred method of communication was Facebook. As a response to these barriers, WebRDS recruitment was introduced. Following approval by the Curtin University Human Ethics Research Committee, the study protocol was amended to recruit participants online in parallel to the continued face-to-face recruitment. Adolescents who expressed interest via Facebook or in response to seeing a flyer or Facebook advertisement had their eligibility to participate confirmed by a research team member before provision of the survey link and a password. The brief screening questionnaire for eligibility and the mature minor assessment were also incorporated into the online survey in multiple-choice format. This process was adapted for the electronic assessment by providing the study information at the start of the survey and subsequently assessing participants’ understanding of the information by asking the same questions as the face-to-face version using a multi-choice format. All questions had to be answered correctly to allow study participation. The process was initially tested face-to-face to ensure its validity.

Contact details of participants were collected at the end of the survey to enable communication with participants for referral purposes and sending incentives in the form of electronic gift cards. Survey responses were checked and transferred to the study database daily and contact details were checked against other participants for duplication. Referral messages were sent to the participant within 24 hours and incentives within 48 hours of survey completion. Referral coupons were provided to the participant via the survey link accompanied by 3 unique identifying passwords within the referral messages. Participants were asked to forward the survey link and passwords to their peers via their preferred social media. The passwords were identical to the referral codes. Passwords expired after use to ensure each code was used only once and to distinguish between participants.

### Recruiting via Facebook

One week after the online referral method commenced, a Facebook business page was created for the study to enhance communication with participants and recruitment. Data continued to be collected via a separate survey Web-hosting site. To increase response rates, we also explored the use of Facebook advertisements and post promotions. The advertising campaign ran for 35 days during which 3 different advertisements targeting Facebook users who lived in Perth aged between 14 and 17 years were piloted. Two of the advertisements used keywords or specification of interest groups (eg, drinking, alcohol, binge drinking, drinking culture, alcohol intoxication, sports). Example advertisements consisted of the following text: “Curtin University Study—Earn up to $45 JB Hi-Fi gift cards by completing the Youth Alcohol Norms online survey.”

Facebook advertisements were not visible to smartphone and tablet users; hence, page post promotions were also used to boost the visibility and reach of status updates. Status updates are messages/images screened on a Facebook user’s news feed, the center column of an individual’s profile where stories are constantly updated from people and pages that they “like” and then follow. A higher rate of expressions of interest was noted during the time when posts were promoted compared to as a response to the advertisements. Consequently, status updates were used as the main form of promotion.

In total, 6 posts were promoted with different picture and text options. A new post was usually created and promoted on a Friday and/or Saturday afternoon and ran for 24 hours. Facebook page metrics indicated that fans (users who liked our page) were most frequently online Fridays to Sundays, increasing the chances of posts being seen. Five of 6 posts targeted adolescents living in Australia who were between the ages of 14 and 17 years with one of these specifically targeted at youth aged 14 and 15 years to increase participation among this group.

General unpaid status updates were also posted weekly on our page timeline (n=11) as a means of engaging with participants to remind them to complete their survey or invite their peers to the study. To enhance recruitment, several youth-focused sports clubs and youth groups agreed to post the study’s recruitment poster on their Facebook page.

**Figure 1:**
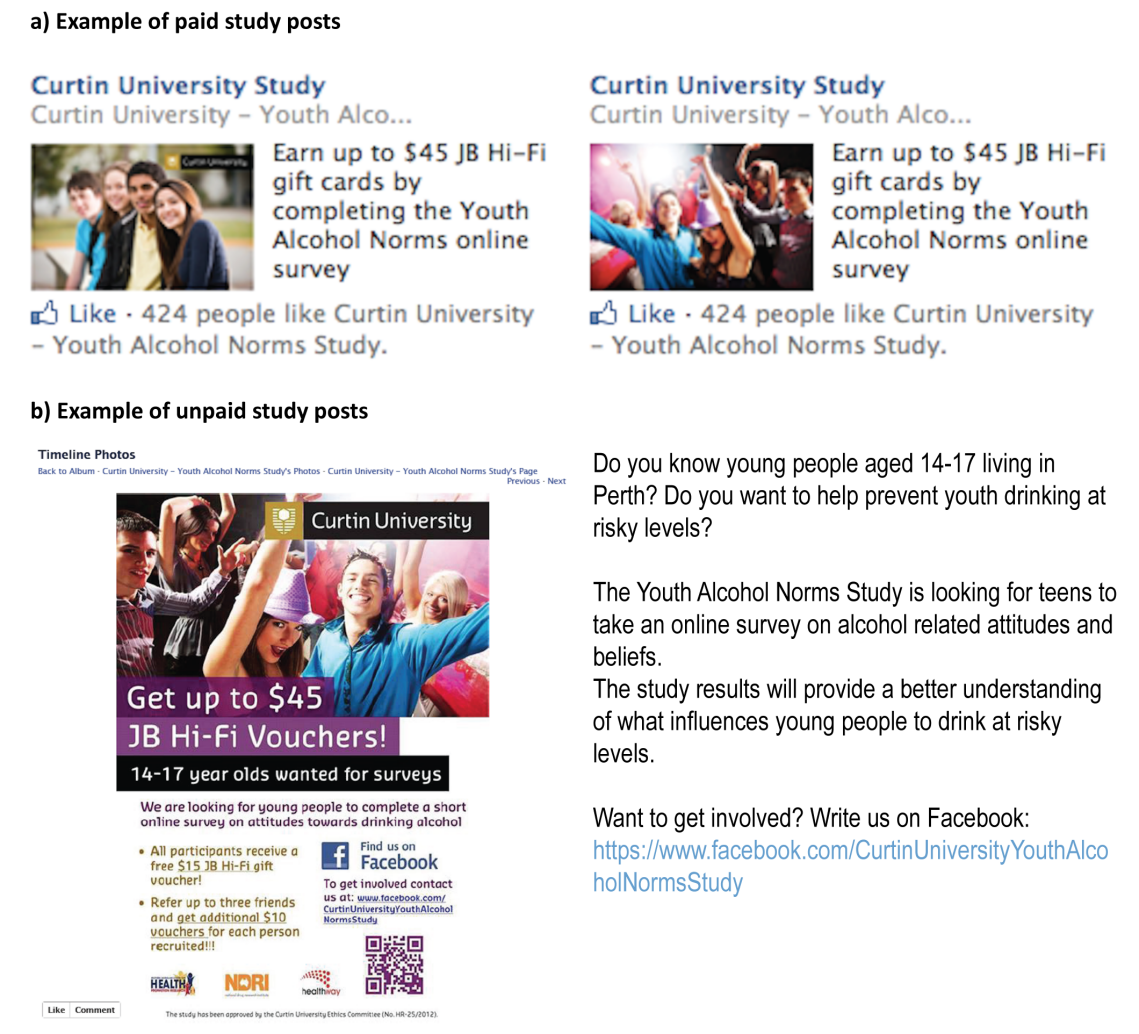
Example of (A) image and text used in study advertisement and (B) unpaid post used during the Facebook campaign.

An example text of a status update included: “Are you aged between 14-17? If YES, then we want YOU for our study! You can earn up to $45 JB Hi-Fi gift cards by simply completing the Youth Alcohol Norms Survey and referring your peers!! Send us a private message to get involved!” An example of 2 images and text typical of a study advertisement and unpaid post used during this campaign are shown in Figure 1.

### Challenges and Solutions of Web-Based Respondent-Driven Sampling

Every effort was made to ensure duplicate survey completion was detected. Participants were initially screened for study eligibility before being able to proceed to the survey. Rapid referrals and similar or identical email addresses and contact numbers to previous participants were investigated. Geodata information about the geographic location of survey completions aided this process to identify respondents who did not meet location criteria. When screening raised questions of eligibility, referral messages and incentives were not sent and participants were contacted by phone, email, or Facebook to verify their identity. Some participants were asked to submit an appropriate form of identification, but there was generally a lack of response or participants were unable to provide the required documentation. Because participants contacted us via Facebook private messaging to receive their coupon codes and their gift cards, we also verified their profile information against the identification documents provided. For those where suspicion was raised or if no identifying information was provided, Facebook accounts were often found to be recently created with no content or friends, confirming a fake profile. If fraudulent behavior was confirmed, the participant was sent a message informing them that they were ineligible to continue to participate in the study or to receive their incentives. If a participant acted as their own referral and completed the survey multiple times, only the first of the duplicated surveys was retained for statistical analysis.

### Data Analysis

Statistical data analyses were performed using SPSS version 21 (IBM Corp, Armonk, NY, USA). Pearson chi-square (χ^2^) test (Fisher’s exact test if applicable) was used to assess the difference in sociodemographic profile and drinking behavior between data collected by RDS and WebRDS techniques. All tests were 2-tailed using a significance level of 5%.

Demographic characteristics (gender, age, indigenous status, main language spoken at home, and socioeconomic status [SES] measured by postcode) and drinking behaviors of the sample recruited via RDS were compared to those sampled by WebRDS. Participants’ socioeconomic level was derived from population data from the 2011 Australian Socio-Economic Indexes for Areas (SEIFA) summary by postal area by Relative Socio-Economic Advantage and Disadvantage. The SEIFA provides population estimates by ranking them on a scale of advantage (high values) to disadvantage (low values) [29].

### Cost Analysis

In calculating the total cost of traditional RDS, conservative estimates of an average of 3 hours per agency or location, with 2 study staff per visit receiving AU $30 per hour, were used. We consider this conservative because liaison with agencies was not incorporated. This liaison occurred for 6 weeks before data collection began and continued during data collection for another 6 weeks. The costs account for travel, estimating an average distance of 30 kilometers to and from each location by each study staff member, reimbursed at a rate of AU 64.5 cents per kilometer. We estimated an additional 30 minutes was spent by paid staff for administration purposes, namely sending 3 sets of follow-up referral messages to each participant recruited by RDS and WebRDS. We estimated this was approximately 300 hours of work, which corresponded to AU $9000 in staff costs. Further, the RDS costs did not include the time spent by study staff members to follow up agencies before visiting or afterwards to arrange additional visits. The expenses for WebRDS included the cost of the Facebook campaign (AU $430.44), with conservative estimates of one full-time equivalent staff member (38 hours per week) employed to monitor data for 8 weeks. Finally, incentives were not costed in this analysis for either method.

## Results

### Respondent-Driven Sampling Seed Recruitment

Using the described methods, a total of 148 organizations were initially contacted, of which 72 were sports clubs and 76 youth programs. Of these, 25 organizations agreed to participate and received presentations facilitated by research staff. Ten seeds were successfully recruited from 9 sports clubs and 11 seeds from 10 youth programs, all of whom completed the survey. In addition, 54 seeds were recruited across 20 community locations and via study promotion flyers or referrals by parents or teachers, with a total of 75 seeds recruited.

### Web-Based Respondent-Driven Sampling Seed Recruitment

A total of 68 seeds were recruited (61 through Facebook and 7 from friends liking the study page or posts).

### Advertising Campaign

To measure the reach of the Facebook campaigns, “impressions” (the number of times an advertisement was displayed to members of the target demographic), “reach” (the number of people who received impressions of an advertisement or page post), and “clicks” (the number of clicks an advertisement received) were recorded. The 3 advertisements, 6 promoted posts, and 11 status updates resulted in a total of 652,522 impressions, a reach of 88,280 adolescents, and 1426 (1.62% of possible accounts reached via the campaign) youth clicked the advertisement or post. Five of the 6 posts were targeted to youth aged 14 to 17 years Australia-wide with only the final post specific to the Perth region. The number of youth who took action in response to an advertisement or page post totaled 1412 (1.6%), which included actions such as page likes, conversations, and post comments. Figure 1 describes this recruitment process. The highest reach was achieved by a post that received 17,641 impressions resulting in 664 response actions. Generally, posts had lower impressions but a higher numbers of clicks, which is likely due to the larger group that could see the posts (Australia-wide) compared to the advertisements (Perth region).

### Recruitment Results

A total of 96 surveys were excluded. These included surveys from respondents who reported the same contact details across multiple survey entries (n=47) and provided their parent’s contact details to do the survey multiple times (n=5). Participants who provided incorrect demographics were also excluded if they did not complete the survey within Perth and participants were unable to verify they lived in Perth (n=21), or respondents who were suspected to have reported a false age and did not provide a form of verification of identity on request (n=23).

**Figure 2:**
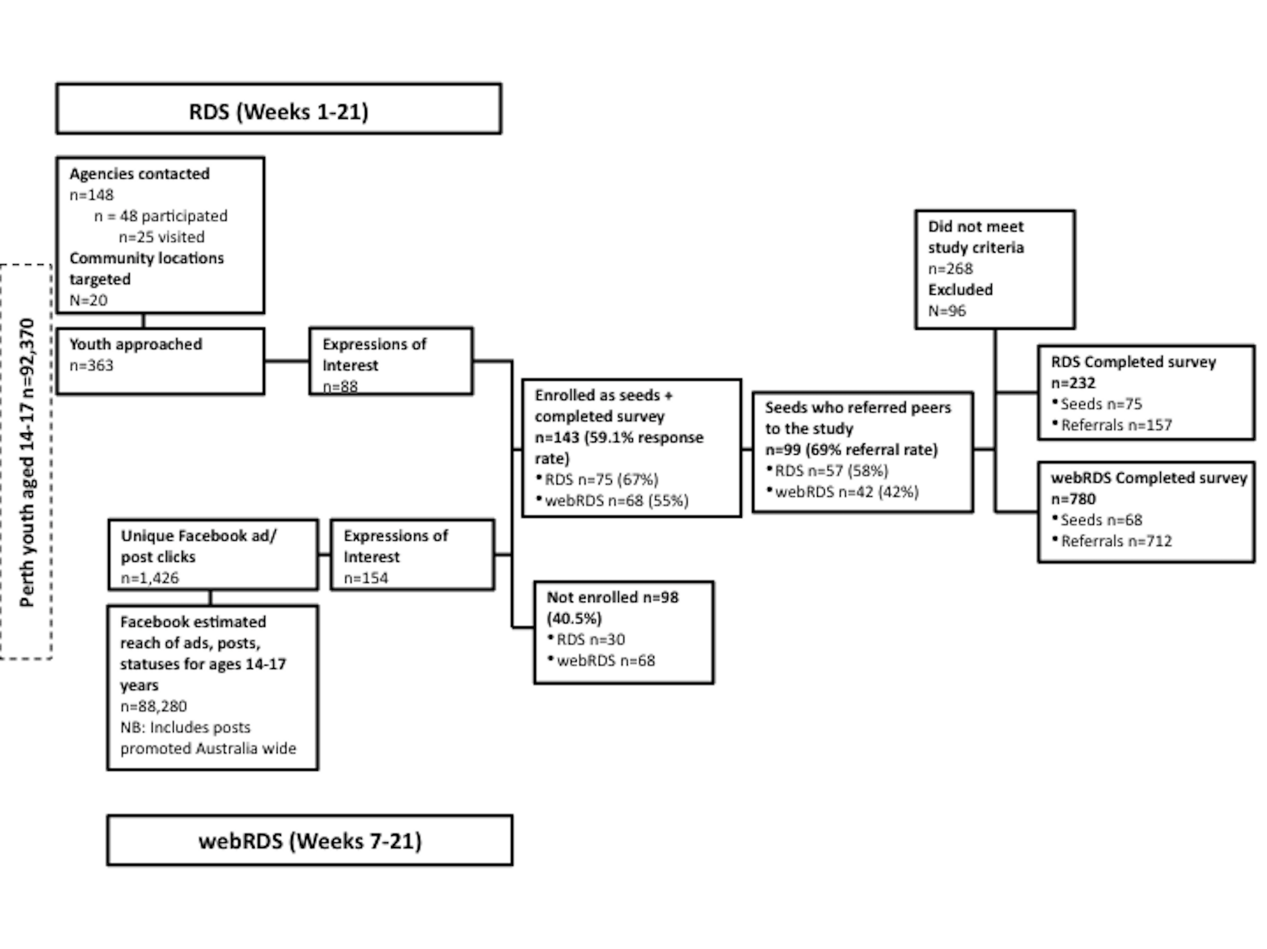
Recruitment process.

A total of 143 seeds (75/143, 52.4% in RDS; 68/143, 47.5% in WebRDS) were recruited and completed the survey in both sampling methods, resulting in 869 valid participants recruited through the referral process (157/869, 18.1% in RDS; 712/869, 81.9% in WebRDS) (Figure 2). Table 1 presents the final sample of valid seeds that completed the survey. Seeds represented 14.13% (143/1012) of the overall sample recruited; 69.2% (99/143) of all seeds recruited made at least one referral and 46.25% (468/1012) of all participants recruited peers to the study.

The mean chain length in this study was 2.3 (SD 2.6), ranging from 0 to 12 waves per chain. When excluding seeds who did not recruit any participants (n=37), the mean chain length was 3.1 (SD 2.5). The majority of chains (n=37) consisted of one recruited wave followed by 2 waves (n=21) and 3 waves (n=12) per chain. In all, 25 chains recruited between 4 and 7 waves, whereas fewer chains (n=7) consisted of waves ranging from 8 to 12. The lengths of RDS and WebRDS chains did not vary significantly (*P=*.14).

**Table 1:** Characteristics of seeds who made a referral (n=99) and total recruited seeds (n=143) by recruitment method.

Age by recruitment method		Seeds, n (%)			
		Seeds who made referral		Total recruited seeds	
		Male	Female	Male	Female
**RDS** ^a^ **(n=57 and n=75)**					
	14 years	7 (12)	5 (9)	4 (10)	2 (5)
	15 years	8 (14)	6 (11)	8 (19)	3 (7)
	16 years	6 (11)	9 (16)	5 (12)	8 (19)
	17 years	6 (11)	10 (18)	8 (19)	4 (10)
**WebRDS** ^b^ **(n=42 and n=68)**					
	14 years	9 (12)	7 (9)	6 (9)	3 (4)
	15 years	9 (12)	11 (15)	11 (16)	8 (12)
	16 years	8 (11)	11 (15)	6 (9)	12 (18)
	17 years	8 (11)	12 (16)	10 (15)	12 (18)
**All (n=99 and n=143)**					
	14 years	11 (11)	7 (7)	15 (10.5)	10 (7.0)
	15 years	16 (16)	9 (9)	20 (14.0)	19 (13.3)
	16 years	11 (11)	17 (17)	14 (9.8)	23 (16.1)
	17 years	14 (13)	14 (14)	18 (12.6)	24 (16.7)

^a^ Participants who (1) were recruited and completed survey face-to-face at sports clubs, youth programs, or community locations; (2) were recruited face-to-face and sent survey link and password following expression of interest to Facebook business page; and (3) expressed interest to Facebook study page or email contact to research staff as a result of seeing a study flyer or being made aware of the study through friends, teachers, or parents.

^b^ Participants who expressed interest on Facebook study page as a result of viewing a study advertisement or a friend’s interaction with the study business page, and who completed the survey on provision of the survey link and a password by the agency coordinator, whom we had liaised with.

**Figure 3:**
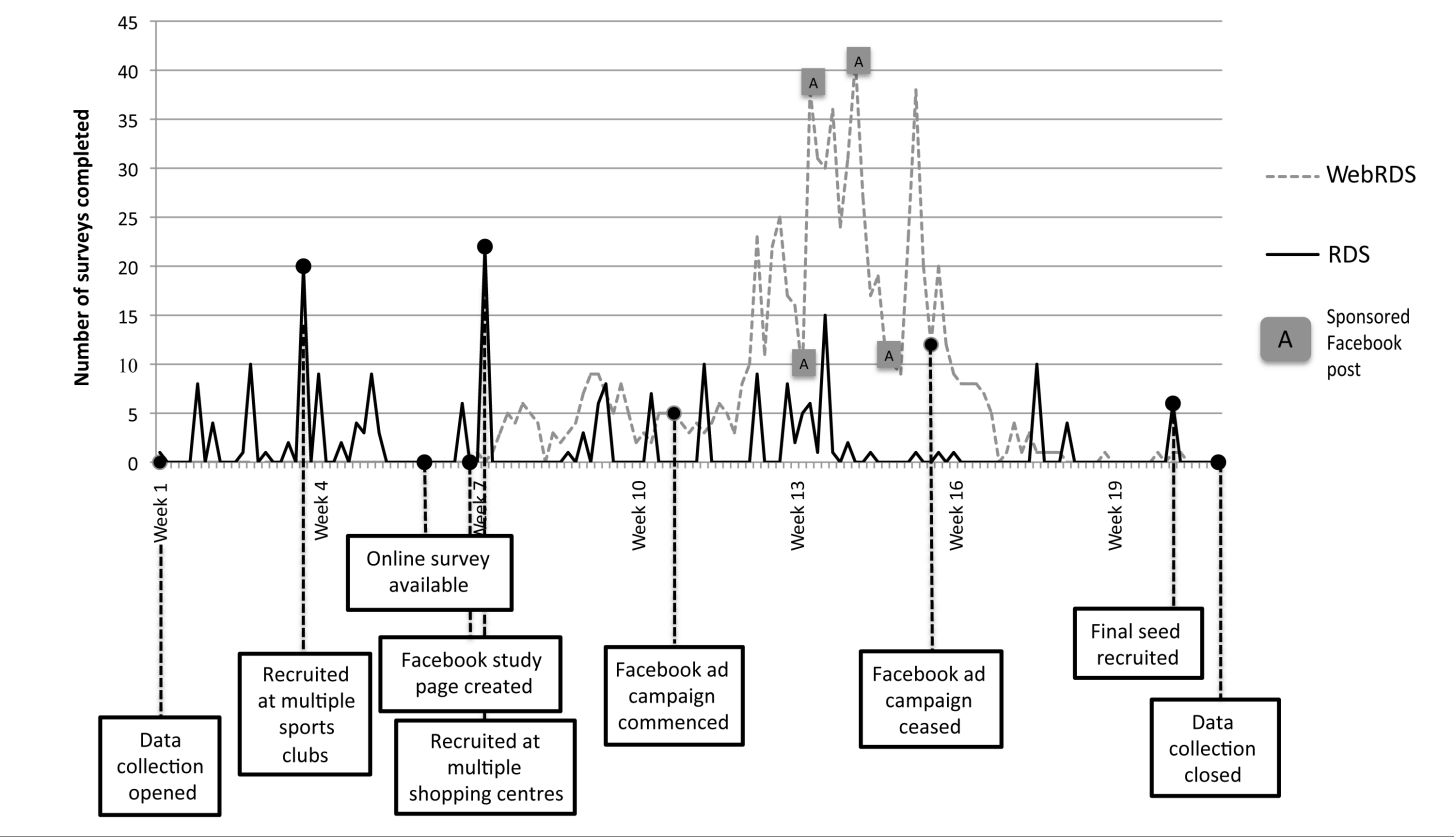
Differences in RDS and webRDS data collection pace.

There was an approximately 5-fold increase in rates of data collection with the transition from traditional RDS to WebRDS (see Figure 3). In the initial 35 days of traditional RDS, a mean of 2.2 (SD 5.0) surveys were completed per day compared to a mean of 8.9 (SD 10.2) surveys per day after WebRDS was launched. The peak daily rate of data collection, which can be attributed solely to completion of online surveys, was 41, which corresponded with the placement of a Facebook advertisement on a Friday. The peak combined rate occurred on the following Monday when 30 surveys were completed online and an additional 15 were recruited by face-to-face recruitment.

### Cost Analysis Results

WebRDS allowed us to boost the number of surveys and speed of data collection by including Facebook posts and advertisements, which resulted in an immediate amplified response. Within RDS, differences were noted regarding recruitment at agencies compared to community locations, the former being more labor-intensive. This was due to the process of initially contacting the agency to establish interest, organizing and conducting the presentation, and then arranging a subsequent time for data collection if it was not feasible immediately after the presentation. In contrast, recruitment at community locations simply involved coordinating a location and time between study staff, conducting brief overviews of the study, and administering surveys with a seed and one or more of their referrals.

Table 2 provides a simple calculation of costs related to each survey method. The average expenses per RDS participant based on the calculations in Table 2 were AU $53.41 compared to AU $27.24 per WebRDS participant.

**Table 2:** Calculation to compare cost per survey of RDS with WebRDS

Cost per recruitment method		Formula	Calculation (AU$)	Total cost (AU$)
**Total cost**				
	RDS	(Number of agencies and locations^a^ visited*mean time spent at each location*number of study staff*cost to pay 1 study staff per hour)+number of agencies and locations^a^ visited*number of study staff*(mean distance traveled to location*cost per km of travel)+number participants recruited by RDS*administration time per participant*cost to pay 1 study staff per hour	(45*3*2*30)+(45*2*30*.65)+(232*0.5*30)	13,335
	WebRDS	Cost of Facebook campaign+(hours of work by 1 staff member*number of staff*cost to pay 1 study staff per hour)+number participants recruited by WebRDS*administration time per participant*cost to pay 1 study staff per hour	430.44+((38*8)*30)+780*0.5*30	21,350.44
**Per participant**				
	RDS	Total cost of RDS/number of participants (seeds and referrals) recruited by RDS	12,390/232	53.41
	WebRDS	Total cost of WebRDS/number of participants (seeds and referrals) recruited by WebRDS	21,250.44/780	27.24

^a^ Number of agencies and locations visited were n=25 and n=20, respectively.

### Participant Description and Drinking Behavior

A total of 1012 (n=232 in RDS; n=780 in WebRDS) valid surveys were included in the analysis. A similar proportion of gender and age groups were recruited in both samples (see Table 3). RDS recruited a significantly higher proportion of youth who identified as Aboriginal or Torres Strait Islander (5.6%, 13/232 RDS vs 0.8%, 6/780 WebRDS, *P*<.001), lower proportions of youth who spoke other languages than English as their main language at home (5.6%, 13/232 RDS vs 10.5%, 82/780 WebRDS, *P=*.03), and tended to recruit more adolescents living in areas of middle and lower SES (33.6%, 78/232 RDS vs 17.4%, 136/780 WebRDS, *P*<.001) compared to WebRDS. Significant differences in some alcohol use characteristics (past 7-day drinking and past 7-day risky drinking) were observed between the sampling methods, but these differences need to be treated with caution due to the low sample sizes.

## Discussion

### Principal Results

This study recruited a sample of 1012 adolescents in the community, of which 232 were recruited by RDS and 780 by WebRDS. In summary, a significantly larger proportion of Aboriginal or Torres Strait Islander (*P*<.001) participants who spoke English as their main language at home (*P=*.03) and of middle and lower SES (*P*<.001) was found in the RDS sample. The RDS sample was also found to have a higher occurrence of past 7-day drinking (*P*<.001) and past 7-day risky drinking (*P=*.004). No significant differences in gender, age, past month alcohol use, and lifetime alcohol use were observed between the RDS and WebRDS samples. This study revealed RDS and WebRDS used similar lengths of chains for recruiting participants; however, WebRDS had a faster rate of recruitment at a lower average cost per participant compared to RDS.

**Table 3:** Demographic characteristics and alcohol prevalence rates for RDS and WebRDS samples.

Demographic variables		Sample population (unweighted), n (%) N=1012^a^		*P*
		RDS sample n=232^b^	WebRDS sample n=780^b^	
**Sex**				.82
	Male	136 (58.6)	465 (59.6)	
	Female	96 (41.4)	315 (40.4)	
**Age (years)**				.10
	14	49 (21.1)	133 (17.1)	
	15	71 (30.6)	207 (26.5)	
	16	58 (25.0)	256 (32.8)	
	17	54 (23.3)	184 (23.6)	
**Indigenous status**				<.001
	Aboriginal or Torres Strait Islander	13 (5.7)	6 (0.8)	
	Not Aboriginal or Torres Strait Islander	215 (94.3)	770 (99.2)	
**Main language spoken at home**				.03
	Australian	218 (94.4)	694 (89.4)	
	Other	13 (5.6)	82 (10.6)	
**Socioeconomic level (SEIFA deciles)**				<.001
	1-3	19 (8.4)	5 (0.7)	
	4-7	59 (26.2)	131 (17.1)	
	8-10	147 (65.3)	630 (82.2)	
**Drinking behavior**				
	Never used alcohol	51 (22.2)	210 (26.9)	.17
	Past month drinking^b^	75 (64.1)	156 (54.4)	.08
	Past 7-day drinking	50 (21.6)	90 (11.6)	<.001
	Past 7-day risky drinking^c^	30 (13.0)	52 (6.7)	.004

^a^ Numbers may not add up to total (N=1012; RDS: n=232; WebRDS: n=780) due to missing data.

^b^ “Past month drinking” only completed by participants who reported having ever drunk before.

^c^ Risky drinking derived from “past 7-day drinking” variable using National Health and Medical Research Council guidelines of >4 drinks per day [30].

### Comparison of Respondent-Driven Sampling to Web-Based Respondent-Driven Sampling

WebRDS represented a considerable advantage over the traditional RDS recruitment method as evidenced by the significant increase in referrals and reduction in cost per participant. It is possible that the slow recruitment rate of traditional RDS was due to the incentive not matching perceived effort and time required, or the intimidating aspects of contacting and meeting unfamiliar study staff. WebRDS reduced staff requirements and was more conducive to the online presence and preference for interaction via social media of the target group. In contrast to phone contact, interaction via Facebook appeared to be easier and possibly less invasive to study participants. Further, WebRDS enhanced anonymity and allowed participants to complete the survey in their own personal space and separate from peers. Most referrals were made in the first 2 weeks following survey completion, supporting the importance for rapid follow-up with participating youth in an appropriate manner to maximize chances of referrals being made.

In this study, we managed to recruit a large number of community-based participants within a short period after exploring various recruitment options. Significant human resources were required to conduct both the RDS and WebRDS recruitment. WebRDS yielded a considerably higher rate of completed surveys in a much shorter timeframe and at approximately half the cost per participant than traditional RDS. This is similar to findings of Wejnert and Heckathorn [24] suggesting that WebRDS has the potential to recruit large study samples up to 20 times faster than traditional RDS. Bauermeister and colleagues [21] also reported an expedited recruitment rate after offering participants the option to refer peers by email, text message, or social network post or message. However, there are differences in the methods implemented that limit direct comparison. The latter study recruited nearly 3500 participants in a 6-week period. This was facilitated by the use of automatically generated coupon codes and referral emails allowing a very quick and efficient response to participants. In contrast, we conducted the process manually, which left some participants waiting up to 48 hours for a response. Further, Bauermeister et al [21] allowed payment for up to 5 referrals with multiple use of referral codes and sampled from various regions across the United States, not just one city.

WebRDS proved to be an efficient method of recruitment and data collection with significant advantages over the RDS in agency and community locations. Notwithstanding, and similar to Bauermeister et al [21], a number of issues arose using this method. WebRDS posed a higher risk for duplicate and falsified surveys during the online recruitment and a substantial amount of time was allocated to screening participants and surveys to verify the data validity. Immediacy of response to potential participants and recruits to mail out survey invitations, referral messages, and incentives was vital to maintain the recruitment momentum and daily data screening and participant follow-up was necessary to ensure the quality of the data. However, this was difficult to achieve during peak times of survey completion.

Other studies using Facebook advertisements and online surveys reported confirming eligibility only as part of their survey [31-33]. However, due to the challenges encountered with online recruitment and confirming a person’s identity, more rigorous procedures were adopted in this study. Although there was a risk of deterring youth from taking part by requesting identification, we included this step to ensure validity of the data. This was particularly important because most of the participants who expressed interest were seeds, which could have determined the quality of data for an entire chain of participants.

Almost half of our sample (46.25%, 468/1012) recruited participants to the study. These results are comparable to other RDS studies and coherent with the geometry reported for RDS recruitment patterns [24]; namely, if each respondent is asked to recruit 3 peers, approximately one-third of participants will make a referral [24]. For example, Thompson et al [34] studied street-involved youth aged 14 to 24 years using RDS; of 156 participants who received referral coupons, 67 (42.9%) recruited at least one peer. Of the 468 respondents who made a referral, 99 represented seeds resulting in 9.8% of the total sample recruited. Other studies have reported varying results with the proportion of seeds in relation to the total sample recruited, ranging from 1.9% [35] to 59.7% [34], possibly owing to the frequently reported difficulties in recruiting active seeds [36,37]. The length of a chain indicates the success of RDS and it has been proposed that social connectedness exists between participants if at least one chain achieves to recruit 3 waves [34]. No differences were found between the number of waves per chain between RDS and WebRDS (*P*=.14) in this study, with the mean length of chains being 2.3 (SD 2.6) waves and a maximum of 12 waves reached per chain. A large proportion of chains consisted of 1 to 2 waves only (n=58); however, we also managed to recruit 12 chains consisting of 3 waves and 32 chains, which achieved 4 to 12 waves, suggesting that the definition of connectedness between participants was met. There is limited information reported on the lengths of chains recruited by other RDS studies with young people; however, the findings suggest that our study recruited substantially more chains with longer waves [14,34,38]. For instance, Thompson et al [34] recruited a total of 17 chains of which only 3 achieved 3 to 4 waves and 2 chains reached 7 and 9 waves, respectively.

In this study, WebRDS and traditional RDS recruited participants with similar proportions of males and females (*P*=.10), of age groups from 14 to 17 years (*P*=.82), and of “never consume alcohol” (*P*=.17) and of “consumed in past month” (*P*=.08). However, traditional RDS recruited more indigenous participants (*P*<.001), fewer non-Australia ethnicities (*P*=.03), and more participants at a lower socioeconomic level (*P*<.001) compared to WebRDS. Traditional RDS participants were also more likely to have consumed alcohol in the past 7 days (*P*<.001) and consumed alcohol at risky levels in the past 7 days (*P=*.004), although these results may not be accurately compared due to the low numbers in these categories.

Research from the United States reported that although nearly all teenagers aged 12 to 17 years use the Internet, those who do not are more likely to be in households with lower income and less access to technology [39]. Higher SES predicts current Internet use and amount of Internet use, with teenagers of higher SES more likely to use the Internet for more time [40] and more likely to use it for social purposes [41] than lower SES teenagers. Congruent with these findings, the WebRDS sample in our research had a higher average SES. If people with higher SES used the Internet more often (assuming this follows with more Facebook use) than those of lower SES, they were more likely to see the Facebook advertisements, posts, and statuses and more likely to receive their online referral. The agencies and community locations in our RDS sample varied with respect to SES with methods in place to ensure distributive representation. This likely contributed to the higher proportion of lower SES youth in RDS than WebRDS, reflecting the increased diversity of this sample. In addition, indigenous Australians are more likely to be lower SES [42] than nonindigenous Australians, which may explain why RDS sampled a higher proportion of indigenous youth than WebRDS.

There are several explanations that could be posited for the differences in participants’ main language spoken at home recruited by the different methods, whereby RDS recruited a smaller proportion of this group. Firstly, it could be because of the type of agencies and locations where recruitment occurred. Australian data show that children younger than 15 years born in non-English-speaking countries or whose parents were both born in non-English-speaking countries participate in organized sport at lower rates than their Australian-born counterparts [43]. Sports clubs accounted for 35% of the agencies visited in this study. In addition, the majority of sporting agencies were Australian Rules Football clubs, a sport primarily played in Australia, with less representation from more global sports such as cricket, gymnastics, and martial arts. Secondly, RDS recruitment was limited in agencies or locations that may have been more culturally diverse, such as at churches and specific cultural events. Finally, research shows that more children younger than age 15 years born in an English-speaking country other than Australia access the Internet than children born in Australia [43]. This is in contrast to what was observed by Bauermeister et al [21] that ethnic minorities use the Internet less than US-born individuals [39,40]. The combination of these factors led to non-English first language speakers being less likely to be sampled in RDS and more likely to be sampled in WebRDS.

Our research targeted specific groups through traditional RDS. There is potential that purposive seed sampling in WebRDS could be used to achieve a similar sample distribution as RDS. This would require more precise eligibility screening of potential seeds to ensure representation of different demographics. This could be achieved through Facebook when the person expresses interest (eg, screening on their age, postcode, and ethnicity). Doing this would come with its own set of challenges and may result in a smaller sample size depending on available resources, which would also limit representativeness. Future research on using the Internet, in particular Facebook, as a recruitment tool needs to focus on the best way of doing so to obtain high validity and reliability with careful consideration of the target population.

### Facebook Recruitment

Facebook was used in this study to recruit participants to supplement conventional data collection methods. Using Facebook to enhance our recruitment resulted in fast response rates and a wide reach, presumably due to being a more acceptable form of communication among youth [44]. This has important implications because previous research has noted barriers to recruiting adolescents for research studies [33]. However, a recent Australian study recruiting young women aged 18 to 23 years via a range of different methods reported Facebook achieved the greatest success, recruiting a cohort of young women with similar characteristics to those of Australian women in terms of age, area of residence, and relationship status [45].

Although young people’s access to the Internet is high in Australia [43], sampling from Facebook could have introduced some biases because the population was limited to adolescents who have access to the Internet, have a Facebook account, provided and matched the demographics (eg, age and location) targeted by our advertisements, and were actively logged into their account while the advertisements were screened. There may also be issues when targeting those with registered “interests” which may be more effective in attracting those interested in the topic [46,47]. Yet, this method could also prove useful when aiming to study subpopulations with specific attitudes and behaviors as was the case in our study.

Interestingly, our advertising campaign achieved a higher rate (1.6%) of youth who took action in response to an advertisement or promoted post in proportion to reach compared to other research using Facebook for recruitment. For example, Kapp and colleagues [31] and Ramo and colleagues [46] reported 0.075% and 0.7% of potential accounts reached via their campaigns resulted in clicks, respectively. These variations may be due to different incentives, target groups and cultures, Internet access behaviors, and that the use of posts in our study may have reached more users than advertisements only.

Due to the peer referral process, we were unable to determine how most participants found out about the study because we were concerned that it would have further increased respondent burden and deterred participants. Thus, in most cases it was not possible to elucidate whether the advertisements/posts participants saw were screened by our campaign or were viral posts from Facebook friends.

### Limitations

Overall, there were difficulties in motivating adolescents to take part in the study. There appeared to be a greater interest in the study among males, which was reflected in their rate of participation. It is possible that the form of incentive was more appealing to males, thus more neutral incentives should be considered in future studies in which gender equality is desired. As part of the overall project objectives, during the RDS, we specifically sampled youth from community programs catering for at-risk youth. In contrast, the lack of specificity using WebRDS may have created over- or undersampling of certain minority groups.

In addition, although there were no significant differences in the length of RDS and WebRDS chains, the chains created by youth recruited in person who referred their peers “on the spot” may have represented different types of connections than those who invited peers via Facebook where any of their Facebook friends could have clicked on the survey link. Hence, the nature of connections between RDS and WebRDS participants may be inherently different in yet unexplored aspects. Due to the difficulties in accurately determining fraudulent activity in WebRDS, it is possible that not all duplicate or falsified surveys were detected. Future research should also note that surveys should be considered on a case-by-case basis. Nevertheless, our findings related to RDS and WebRDS recruitment contribute to the literature and provide a reference for others intending to conduct similar studies.

### Conclusions

There is a need to constantly improve the quality of Web-based surveys [48]. This is one of the first papers to describe the processes undertaken to gain 2 samples using both traditional RDS and WebRDS. Overall, Facebook was the most successful recruitment source for adolescents to complete an online survey compared to face-to-face recruitment and other forms of online recruitment and referral. A factor that likely contributed to this is the increasing preference of social networking sites for communication purposes among youth, which reduce the barriers to participation than more traditional recruitment methods. Although we were successful in using Facebook as a recruitment strategy, it is still a novel method and more research is necessary to overcome associated challenges and minimize biases. WebRDS requires continual monitoring and cleaning of data to screen suspicious participants. Such monitoring and the need for quick responses can be challenging, particularly if all communications and generation of referral codes is done manually. However, WebRDS allowed for a faster rate of recruitment at a lower average cost per participant than traditional RDS. WebRDS increased the ease of informing the target population about the study and is particularly useful for recruiting populations, which are traditionally difficult to access. Measures need to be in place to ensure the demographics of WebRDS match traditional RDS, which could be done by purposive seed selection in both methods. Many other popular social networking sites exist that other population groups may access in preference to Facebook and the key is to ask the desired target group their preferences. The experience of this study does not just promote Facebook as a recruitment tool, but is a cue to exploring social networking sites as a means of recruitment.

## Abbreviations

RDS: respondent-driven sampling
SEIFA: Socio-Economic Indexes for Areas
SES: socioeconomic status
WebRDS: Web-based respondent-driven sampling

## Appendix

Multimedia Appendix 1 Application of the Checklist for Reporting Results of Internet E-Surveys (CHERRIES)[48] to the Youth Alcohol Norms Survey (YANS).
