# Supplementary material for: Potential and Challenges in Collecting Social and Behavioral Data on Adolescent Alcohol Norms: Comparing Respondent-Driven Sampling and Web-Based Respondent-Driven Sampling
Source: J Med Internet Res. 2015 Dec 24;17(12):e285. doi: 10.2196/jmir.4762 (PMC4705369; doi:10.2196/jmir.4762)
Supplement: Multimedia Appendix 1 [file jmir_v17i12e285_app1.pdf]

Supplemental Table 1. Application of the Checklist for Reporting Results of Internet E-Surveys (CHERRIES)[48] to the Youth Alcohol Norms Survey (YANS)

Item Category	Checklist Item	YANS Survey
**Design**		
	Survey design	Convenience sample.<br>14-17 year old community-based adolescents in Perth, Western Australia.
**IRB (Institutional Review Board) approval and informed consent process**		
	IRB approval	Received ethical approval from the Curtin University Human Research Ethics Committee (approval number: HR-25/2012)
	Informed consent	Informed consent was sought by employing a mature minor assessment, explaining to participants of the purpose of the study, who was conducting it, the anticipated outcomes, that all data was stored and treated confidentially, along with the requirements of participation including length of survey time and how to refer peers to the study. It was explicitly stated that the decision to participate in the research was that of the young person's alone and that they could withdraw at any time without prejudice.
	Data protection	Confidentiality procedures included:<br>(1) Delinked data collection: all data collected remain anonymous by removing personal identifiers from the database following verification of participants' identity<br>(2) Direct entry of de-identified data into the computer system:

Item Category	Checklist Item	YANS Survey
		software removed the need for an interviewer to observe the responses<br>(4) Data storage: after each interview the data was encrypted and downloaded on a secure computer with password protection on a PC in a lockable office at the research institution<br>(5) Training: All project personnel received training in confidentiality protection before data collection to ensure maximum participant protection.
**Development and pre-testing**		
	Development and testing	The instrument development was guided by Social Cognitive Theory and comprised a systematic multistage process including evaluation of content and face validity. Using a two-week test re-test format, the YANS was administered to secondary school students (n = 481, age = 13–17 years, female 309, 64.2%) followed by analysis of exploratory factor analysis, correlations between extracted factors and socio-demographic, internal consistency and test-retest reliability.<br>The electronic version of the survey was tested with members of the target group for feasibility before implementation.
**Recruitment process and description of the sample having access to the questionnaire**		
	Open survey versus closed survey	Closed survey – password protected

Item Category	Checklist Item	YANS Survey
		participants was made face-to-face (RDS), through which 75 seeds were recruited. Following adoption of the web-based approach (webRDS), initial contact predominantly changed to the Internet.
	Advertising the survey	Announced by youth program/sports group facilitators/coaches who circulated a flyer via mailing lists.<br>In person, explaining the study and participation requirements verbally.<br>Facebook advertisement (see Figure 1 for details).
**Survey administration**		
	Web/E-mail	Sent via email – responses were manually downloaded into the database
	Context	No website was used to distribute the survey
	Mandatory/voluntary	The nature of the study and survey completion was entirely voluntary
	Incentives	Participants were offered a AUS$15 electronic store gift voucher for survey completion, and a maximum of 3 AUS$10 gift vouchers for making a successful referral to the study
	Time/Date	21 weeks
	Randomization of items or questionnaires	Not applicable
	Adaptive questioning	The survey used adaptive questioning to reduce number and complexity of the questions
	Number of Items	2 to 11 items per page

Item Category	Checklist Item	YANS Survey
	(pages)	
	Completeness check	No – responses were not enforced due to working with minors to allow them the choice to respond
	Review step	No
**Response rates**		
	Unique site visitor	This data was not collected
.	View rate (Ratio of unique survey visitors/unique site visitors)	This data was not collected
	Participation rate (Ratio of unique visitors who agreed to participate/unique first survey page visitors)	This data was not collected
	Completion rate (Ratio of users who finished the survey/users who agreed to participate)	1376 people agreed to participate and completed the last page of the survey. 907 people submitted the first page but did not complete the survey.
**Preventing multiple entries from the same individual**		
	Cookies used	Cookies were used on the first page of the survey to assign a unique user identifier to each client computer.<br>Users were both prevented by preventing access to the survey twice through use of individual passwords, and duplicate entries with the same user ID were removed from the database prior to analysis, keeping the first entry for analysis.
	IP check	The IP address of the client

Item Category	Checklist Item	YANS Survey
		computer was not collected
	Log file analysis	Not applicable
	Registration	Participants received a unique referral code in form of a password which expired after use. Once the survey was completed, the survey could not be accessed again.
**Analysis**		
	Handling of incomplete questionnaires	Only completed questionnaires were analyzed
	Questionnaires submitted with an atypical timestamp	No cut off point was used
	Statistical correction	Not applicable
